# Repeated Episodes of GPP Induced by Respiratory Infections Are Mediated by Loss‐of‐Function *IFIH1*

**DOI:** 10.1002/iid3.70397

**Published:** 2026-03-18

**Authors:** Yaqin Liu, Yanan Sun, Juan Yang, Hongmei Li, Weihui Zhou, Shasha Meng

**Affiliations:** ^1^ Department of Pediatric Research Institute, National Clinical Research Center for Children and Adolescents' Health and Diseases, Ministry of Education Key Laboratory of Child Development and Disorders, Chongqing Key Laboratory of Child Neurodevelopment and Cognitive Disorders Children's Hospital of Chongqing Medical University Chongqing China; ^2^ Shenzhen Traditional Chinese Medicine Hospital Shenzhen Guangdong China; ^3^ Department of Dermatology Children's Hospital of Chongqing Medical University Chongqing China; ^4^ Center for iPS Cell Research Children's Hospital of Chongqing Medical University Chongqing China

## Abstract

**Background:**

Generalized pustular psoriasis (GPP) is a severe and recurrent autoinflammatory disease that can be induced by a variety of factors. At present, its etiology and pathogenesis have not yet been fully elucidated. Upper respiratory tract infections are the main predisposing factor in children with GPP, and the MDA5 protein encoded by the *IFIH1*, functions as a key receptor for sensing upper respiratory tract viruses and may contribute to the induction of antiviral and immunological responses.

**Methods:**

We performed whole‐exome sequencing in 80 pediatric GPP patients and conducted rare variant association analyses against healthy controls. Structural and expression analyses of mutant MDA5 were performed to evaluate mechanistic consequences. Identified *IFIH1* variants were functionally characterized using IFN‐β luciferase reporter assays, circular dichroism spectroscopy, and protein stability assays.

**Results:**

We identified eight *IFIH1* rare variants in 10 pediatric GPP patients, accounting for 12.5% of the total cohort. Association analyses revealed a significantly higher prevalence of rare variants of *IFIH1* in GPP patients as compared to healthy controls. *IFIH1* variation was found to cause structural changes or decreased expression of its encoded protein MDA5, the intrinsic stability of the mutant was lower than that of the wild‐type *IFIH1*. Notably, functional assays demonstrated that these *IFIH1* variants (6/8) substantially impaired the production of interferon beta (IFN‐β).

**Conclusion:**

This study suggests that loss‐of‐function variants in *IFIH1* may be associated with the pathogenesis of recurrent episodes of GPP triggered by URTI. These findings offer a theoretical basis for the development of targeted etiological and pathogenetic preventive measures.

## 1 Introduction

Generalized pustular psoriasis (GPP) is a subtype of pustular psoriasis characterized by generalized eruption and sudden onset of erythema and sterile pustules. Patients experience recurrent widespread pustular outbreaks, often accompanied by high fever, chills, joint pain, and general weakness; some patients also experience elevated liver enzymes and hypoalbuminemia. Its course is typically characterized by recurrent episodes and a poor prognosis [1, 2]. In pediatric populations, GPP tends to have a more acute onset, with frequent recurrence, occurring multiple times per year or every few years. Although rare in children, this condition frequently presents as a severe, life‐threatening disorder [3, 4, 5]. The etiology and pathogenesis of GPP are not fully understood. However, factors such as genetics, infection, immune abnormalities, and endocrine disorders have been implicated in its pathogenesis [2].

GPP first described by von Zumbusch in 1909 [6], remained etiologically obscure until the discovery of *IL36RN* mutations in 2011 [7, 8]. Loss‐of‐function mutations in *IL36RN* impair interleukin‐36 receptor antagonist (IL‐36Ra) activity, driving dysregulated IL‐36 signaling. This hyperactivation promotes overexpression of neutrophil chemokines (IL‐6, IL‐8) and pro‐inflammatory cytokines, culminating in epidermal neutrophil infiltration, sterile pustule formation, and tissue damage [7]. In addition to *IL36RN* mutations [9, 10, 11], genetic variants in *CARD14* [12, 13, 14], *AP1S3* [15, 16], *MPO* [17], and *SERPINA3* [18, 19] have also been implicated in GPP pathogenesis. However, these known mutations only account for a fraction of GPP cases, particularly sporadic cases or those within specific populations. Researchers have suggested that GPP may not be attributed to a single genetic mutation but rather to the cumulative effects of multiple genes and environmental factors [20].

The onset of GPP is often associated with triggering factors, including infections, drug exposure, stress or corticosteroid withdrawal. In pediatric populations, upper respiratory tract infection (URTI) dominate as the primary precipitating factor, with studies reporting URTI‐associated triggers in 53%–59% of cases [21, 22]. Upper respiratory tract infection (URTI) refers to an infection that affects the upper portion of airway. Symptoms include fever, cough, Sneezing, runny nose or blocked nose, slight body aches or headache. The heightened susceptibility may reflect developmental vulnerabilities to viral pathogens. During viral infections, pathogen‐associated molecular patterns (PAMPs) and pattern recognition receptors (PRRs) trigger signal cascades, ultimately leading to the production of various host defense molecules, including type I/III interferons, proinflammatory cytokines, and chemokines, and the expression of innate immune genes; this promotes intracellular antiviral capabilities [23, 24].

The *IFIH1* encodes the protein MDA5 (Melanoma differentiation‐associated protein 5), which is a cytoplasmic PRR for certain aberrant RNAs, inducing a type I interferon response. MDA5 is a member of the retinoic acid‐inducible gene I‐like receptors (RLRs) family and is capable of recognizing certain viral‐derived ssRNA, dsRNA, and dsDNA, as well as dsRNA analog Poly(I:C) [25, 26]. Basally expressed at low levels in most cells, MDA5 is rapidly upregulated during viral infection. When MDA5 recognizes and binds to a virus, its CARDs domain is released and assembled with the adapter mitochondrial antiviral signaling protein (MAVS) into prion protein‐like aggregates, allowing the downstream effector TNF receptor‐associated factors to cluster into an active “signaling body.” The subsequent signaling cascade induces phosphorylation and nuclear translocation of the key innate immune transcription factors IRF3 and IRF7, and the activation of NF‐κB, thereby inducing the expression of type I and III interferons, innate immune genes, and proinflammatory cytokines and chemokines; thereby further limiting viral infection [27]. Autocrine and paracrine IFN stimulation subsequently induces the transcription of hundreds of IFN‐stimulated genes (ISGs). Some of the proteins encoded by ISGs have direct antiviral functions [28]. In 2017, Asgari, S. et al. reported that MDA5 effectively limits the replication of human RSV and rhinovirus, and MDA5 deficiency causes primary immunodeficiency with extreme susceptibility to common respiratory RNA viruses [29]. Therefore, we hypothesized that *IFIH1* defects, together with variants in other GPP‐causing genes, may establish a genetic susceptibility that facilitates the onset of GPP triggered by URTI.

In this study, rare variants of *IFIH1* were identified in children with GPP by whole exome sequencing. Clinical data were retrospectively analyzed and the results indicated that children with GPP had URTI before GPP onset. It was hypothesized that *IFIH1* variants might interact with variations in *IL36RN* or other genes, potentially contributing to the development of GPP following URTI.

## 2 Methods

### 2.1 Participants

A total of 80 child patients with GPP alone were recruited for this study. The inclusion criteria were as follows: (1) age < 18 years; (2) first episode of GPP without a previous history of PV; (3) differential diagnosis and exclusion of acute generalized exanthematous pustulosis (AGEP) [1]. The diagnosis was made by at least two experienced dermatologists. All subjects or legal guardians provided informed consent before participating in the study. This study was approved by the medical ethics committee of the Children's Hospital of Chongqing Medical University and was conducted according to the principles of the Declaration of Helsinki.

### 2.2 Whole Exome Sequencing and Analysis

The participants' peripheral blood genomic DNA was extracted by iGeneTech for whole exome sequencing. Each individual's 250 ng genomic DNA was sheared using the Biorupter from Diagenode, Belgium, to obtain 150–200 bp DNA fragments. The DNA fragments were end‐repaired, and Illumina Adapter (Fast Library Prep Kit v2.0, iGeneTech) was added. After constructing the sequencing library, AIExome Human Exome Panel V3‐Inherit was used to capture the whole exome for sequencing on the Illumina platform (Illumina, San Diego, CA) with 150 bp paired‐end reads. Adapter sequences and low‐quality bases were trimmed using Trimmomatic. Subsequently, the clean reads were mapped to the reference genome GRCh37 using BWA. We then computed a standard set of post‐alignment quality metrics per sample, including target rate, target mean depth, coverage rate (10× and 20×). PCR duplicates were marked and removed using samblaster. Base quality score recalibration (BQSR) and local realignment were performed using the Genome Analysis Toolkit (GATK). The high‐confidence variant set was annotated for functional consequences and population frequencies using ANNOVAR and snpEff. Variant validation was performed with Sanger sequencing of PCR products amplified from the *IFIH1* from each subject's genomic DNA with the corresponding primer sets in Table S1.

### 2.3 Plasmid Construction

IFN‐β promoter‐driven firefly luciferase reporter plasmid was constructed as described previously. Human *IFIH1* CDS (NM_022168.4) was synthesized and cloned into the expression vector pcDNA3.1 + /C‐(K)‐DYK by the Genscript company. The *IFIH1* mutated constructs were subjected to in vitro mutagenesis with a MutanBEST Kit (TaKaRa). All constructs were determined by Sanger sequencing. The numbering of all *IFIH1* mutations in this manuscript is based on RefSeq NM_022168.4. For rare, nonannotated missense variants, constructs were generated with the mutant allele. For polymorphisms (rare and common), constructs were generated as follows (the allele and amino acid used are listed in parentheses): rs147278787(c.229 C > T, p.R77W); rs183412282(c.1041 A > C, p.L347F); rs117608083(c.1093 A > G, p.K365E); rs369245661(c.1590 C > G, p.N530K); rs550930413(c.1899G>T, p.E633D); rs185928139(c.2115 A > C, p.R705S); rs201142986(c.2232 T > A, p.F744L); rs764489663(c.2601 G > C, p.E867D). All the primers were shown in supplementary (Table S1).

### 2.4 Western Blot Analysis

Cells were plated in a six‐well plate and were co‐transfected as described above with 3 µg of *IFIH1* wild‐type or mutated plasmids and 0.2 ug of pEGFP in each well. The cells were harvested 48 h after transfection and were lysed in 100 µl of radioimmunoprecipitation assay (RIPA) Doc buffer (1% Triton X‐100, 1% sodium deoxycholate, 0.1% sodium dodecyl sulfate, 0.15 M NaCl, 0.05 M Tris‐HCl, pH 7.2) supplemented with protease inhibitors (Roche). The cell lysates were separated on a 4%–10% sodium dodecyl sulfate‐Tris‐tricine gel, and immunoblotting was performed using standard procedures. MDA5 (D74E4) Rabbit mAb (CST) was used to detect MDA5 expression. Monoclonal anti‐β‐Actin antibody produced in mouse clone AC‐15 (Sigma) and Anti‐GFP antibody (Beyotime) were used to detect β‐actin and the GFP protein, respectively. IRDye 800CW‐labeled goat anti‐mouse and IRDye 700‐labeled goat anti‐rabbit antibodies (LI‐COR Biosciences) were used as secondary antibodies. Visualization was performed on an Odyssey system (LI‐COR Biosciences).

### 2.5 Interferon Reporter Assay

HEK293T cells were maintained in 48‐well plates in Dulbecco's modified Eagle medium (Gibco) supplemented with 10% fetal calf serum and 1% penicillin/streptomycin. At 80% confluence, the cells were co‐transfected with pcDNA3.1^+^/C‐(K)‐DYK plasmids encoding MDA5 (wild type) or mutants (25 ng), IFN‐β promoter‐driven firefly luciferase reporter plasmid (100 ng), and a constitutively expressed Renilla luciferase reporter plasmid (p3RLuc, 30 ng) using Lipofectamine 2000 (Invitrogen), according to the manufacturer's protocol. The medium was changed at 6 h post‐transfection, and the cells were subsequently stimulated with 10 µg/mL HMWpoly I:C (Invivogen) using Lipofectamine 2000. The cells were lysed 24 h post‐stimulation, and IFN‐β promoter activity was measured using a Dual Luciferase Reporter assay (Promega, E1910) and GloMax 20/20 Luminometer at DLR‐O‐INJ. All reporter assays were performed with a minimum of three independent experiments, each carried out in triplicate (*n* ≥ 3). Firefly luciferase activity was normalized to the corresponding Renilla luciferase activity for each well to account for variations in transfection efficiency and cell viability. The normalized luciferase activity of the empty vector control group was set as 1 (fold induction), and the data from other experimental groups are presented relative to this value. Error bars represent the standard deviation of three to five independent experiments.

### 2.6 Bioinformatics Analysis of *IFIH1* Mutations

The web‐based ConSurf program (version 2016) was applied to compute the sequence conservation scores in each amino acid reconstitution of three mutations (http://consurf.tau.ac.il/). The protein stability changes were analyzed by the DUET web server using 4GL2 (PDB code) as the reference structure (http://biosig.unimelb.edu.au/duet/stability). The predicted folding free energy change (ΔΔG) upon mutation was quantified. The pathogenicity of variants was predicted by VarCards, which is an integrated genetic and clinical database for coding variants in the human genome (VarCards (genemed. tech)). VarCards provides an intuitive interface of necessary information for researchers to prioritize candidate variations and genes.

### 2.7 Statistical Analysis

The data were analyzed with SPSS or GraphPad Prism software. Quantitative data are expressed as the mean ± SD. Multiple group comparisons were performed with one‐way ANOVA followed by post‐hoc Dunnett's test. Differences in the allele frequencies of *IFIH1* variants between groups and the associations between *IFIH1* variants and GPP were examined with Fisher's exact test. A *p* < 0.05 was regarded as statistically significant.

## 3 Results

### 3.1 Study Participants and Analysis of Exome Sequencing

**Figure 1:**
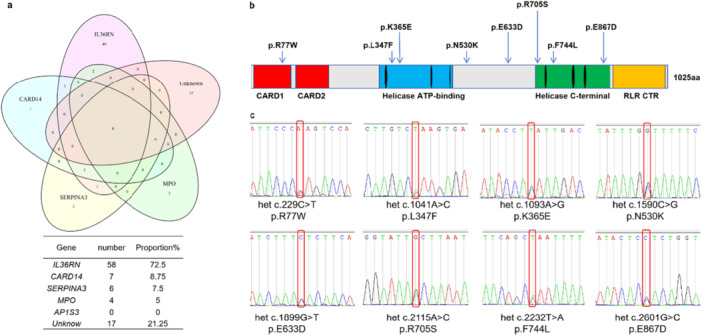
Genetic variant distribution in 80 pediatric GPP Patients and variants identified in *IFIH1*. (a) The Venn diagram illustrates the overlapping relationships between five known disease‐associated genes (*IL36RN*, *CARD14*, *AP1S3*, *MPO*, and *SERPINA3*) and a set of unknown genes (those not yet definitively linked to the disease). Variants in *IL36RN* (72.5%, *n* = 58) predominated, followed by *CARD14* (8.75%, *n* = 7), *SERPINA3* (7.5%, *n* = 6), *MPO* (5%, *n* = 4), unknown (21.25%, *n* = 17). No *AP1S3* mutations were detected. (b) Schematic representation of *IFIH1* mRNA and MDA5 protein domains and locations of Amino Acid Substitutions Missense variants. (c) DNA Sanger sequencing of the 8 variants identified in *IFIH1*. The mutation site of het c.1093 A > G were detected in three patients. DNA was extracted from the whole blood of 10 GPP patients, and PCR amplification was performed on the fragment containing the *IFIH1* mutation site, followed by validation of the mutation site using Sanger sequencing.

A total of 80 Chinese juvenile patients with GPP were recruited for this study after obtaining ethics approval and informed consent. The cohort comprised 48 male and 32 female patients with a mean age at disease onset of 3.9 years. All patients exhibited erythema and aseptic pustules (100%), while 37.5% reported concurrent arthralgia and asthenia (Table S2). To rapidly identify rare variants in the *IFIH1* in GPP patients, a search for disease‐causing mutations was conducted by means of whole exome sequencing. Plasma was isolated from the whole blood samples of simplex GPP patients and DNA was extracted. Successful library construction was achieved for the DNA samples of all 80 GPP patients. Then, whole exome sequencing was performed. All samples individually exceeded the quality threshold, with > 90% of exonic bases covered at ≥ 10×. (Table S3). Whole‐exome sequencing analysis of 80 pediatric GPP patients identified with a high prevalence of genetic susceptibility variants, 63 patients (78.75%) harbored at least one pathogenic variant in the previously implicated GPP‐related genes. Strikingly, *IL36RN* variants accounted for 72.5% (58/80) of the cohort, but among the *IL36RN*‐positive cases, 15% (9/58) of *IL36RN* variant carriers exhibited additional heterozygous mutations in secondary modifier genes, suggesting potential epistatic interactions that may influence disease severity or penetrance. 6.25% (5/80) of patients who carried variants exclusively in non‐*IL36RN* loci in *CARD14*, *MPO* and *SERPINA3*. Noticeably, 21.25% (17/80) of patients lacked detectable variants in established GPP related genes, implicating novel genetic or epigenetic mechanisms in disease pathogenesis (Figure 1a).

Variants were screened if the variant was located in the exonic or splicing region of the gene, with a minor allele frequency (MAF) of less than 0.01 in at least one of the East Asian populations in the 1000 Genomes, ExAC, and gnomAD databases. Strikingly, a total of eight rare variants of *IFIH1* were identified in the 80 pediatric GPP patients. Three patients had the same heterozygous missense mutation c.1093 A > G (p.K365E). The other seven variants were all heterozygous missense mutations: c.229 C > T (p.R77W), c.1041 A > G (p.L347F), c.1590 C > G (p.N530K), c.1899G>T (p.E633D), c.2115 A > C (p.R705S), c.2232 T > A (p.F744L), c.2601 G > C (p.E867D) (Figure 1b). Ten GPP cases were found to have missense mutations in the *IFIH1*, along with eight SNP loci, accounting for 12.5% (10/80) of the total cases. This is significantly higher than the mutation rate of 4.58% in the East Asian population in the gnomAD database (Table S4). Sanger sequencing confirmed the presence of variants in *IFIH1* among 10 GPP patients and all variants were heterozygous (Figure 1c).

### 3.2 Association Analysis Between GPP and the Variants of *IFIH1*

To explore the relationships between these variants and the incidence of GPP, we first compared the differences in the frequencies of these variants between patients and the general population and analyzed the associations between these variants and the disease.

In terms of the frequency of these variants in the general population, the East Asian gnomAD v2 data in the Genome Aggregation Database (https://gnomad.broadinstitute.org) were used. GnomAD v2 contains a very large amount of exome sequencing data that greatly increases its power as a reference in coding region analyses. The allele frequencies of each of the eight variants in the cases were 0.63 to 1.88 times higher than those of the corresponding variants in the control group (Table 1). The combined frequency of the eight variants was 6.25% in GPP patients and 2.30% in controls (Table 1). A statistically significant association between *IFIH1* variants and GPP patients was observed when comparing the GPP group with controls (*p* = 0.004), and *IFIH1* variants as a risk factor for GPP could be established based on the odds ratio (*OR*) and confidence interval (*CI*) (*OR* = 2.84;95% *CI* = 1.49–5.44) (Table 1). Although the results of the association analysis are positive, they should be interpreted with caution, as the control group comes from an external database and may not match in age and gender.

**Table 1:** The association between the *IFIH1* variant alleles and GPP.

*IFIH1* variants	Genotype	aa	Aa	AA	Total	VaFre (%)	*p* (vs. Con)	OR	CI (95%)	Bonferroni correction *p*
c.229 C > T (p.R77W)	GPP	0	1	159	160	0.63	0.429	1.81	0.25–13.13	1
	CON	0	69	19,885	19,954	0.35				
c.1041 A > C (p.L347F)	GPP	0	1	159	160	0.63	0.161	5.96	0.80–44.58	1
	CON	0	21	19,903	19,924	0.11				
c.1093 A > G (p.K365E)	GPP	0	3	157	160	1.88	0.11	2.66	0.84–8.44	1
	CON	1	142	19,775	19,918	0.71				
c.1590 C > G (p.N530K)	GPP	0	1	159	160	0.63	0.017	115.53	7.19–1855.14	0.1
	CON	0	1	18,369	18,370	0.01				
c.1899G > T (p.E633D)	GPP	0	1	159	160	0.63	0.401	1.99	0.27–14.40	1
	CON	0	63	19,879	19,942	0.32				
c.2115 A > C (p.R705S)	GPP	0	1	159	160	0.63	0.477	1.56	0.22–11.29	1
	CON	0	80	19,858	19,938	0.40				
c.2232 T > A (p.F744L)	GPP	0	1	159	160	0.63	0.42	1.87	0.26–13.53	1
	CON	0	67	19,883	19,950	0.34				
c.2601 G > C (p.E867D)	GPP	0	1	159	160	0.63	0.077	13.85	1.75–110.00	0.7
	CON	0	9	19,825	19,834	0.05				
Combined	GPP	0	10	150	160	6.25	0.004	2.84	1.49–5.44	0.04
	CON*	1	452	19,275	19,728	2.30				

*Note:* Differences in frequencies of *IFIH1* variants between groups were analyzed by Fisher exact tests. Bonferroni corrected *p*‐values for some mutation sites are greater than 1. Considering the overall mutation rate, the mutation rate of the *IFIH1* gene in the GPP population may still be higher than that in the general population. Arginine (R), Asparagine (N), Aspartic acid (D), Glutamic acid (E), Leucine (L), Lysine (K), Phenylalanine (F), Serine (S), Tryptophan (W). GPP: generalized pustular psoriasis (*n* = 80); CON: control; aa, homozygote; Aa, heterozygote; AA, wild type; *P*: *p*‐value; VaFre: Variant Frequency; *OR*: odds ratio; *CI*: confidence interval; Bonferroni correction *P*: Bonferroni correction *p*‐value.

* The control data is the allele numbers corresponding variants in the east Asian population adopted from Genome Aggregation Database (https://gnomad.broadinstitute.org/); combined controls' alleles (19,728) are the average of the 8 *IFIH1* variants.

**Figure 2:**
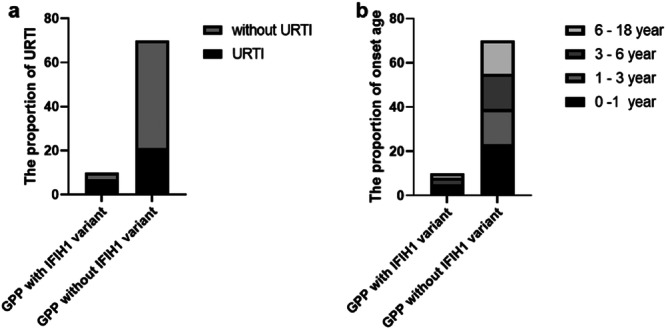
The difference between GPP with and without *IFIH1* variants. (a) The proportion of URTI in GPP with or without *IFIH1* variants. It showed that 7 in 10 URTI in the group of *IFIH1* variants and 21 in 70 URTI in the group of GPP without *IFIH1* variant. The differences in frequencies of URTI between groups were analyzed using Fisher's exact test. *p* ＜ 0.01. (b) The proportion of onset age in GPP with or without *IFIH1* variants. In the *IFIH1* variant group (*n* = 10), there were 4 cases with onset age less than 1 year, three cases had onset age between 3 and 6 years old, and 3 cases had onset age greater than 6 years. In the *IFIH1* non‐variant group (*n* = 70), there were 23 cases with an onset age less than 1 year, accounting for 32.86% (23/70). The number of cases with onset ages between 1 and 3 years and 3–6 years were both 16, accounting for 22.86% (16/70) each. There were 15 cases with an onset age between 6 and 18 years, accounting for 21.43% (15/70). There was no statistical difference between the with or without *IFIH1* variant groups in terms of the rate with an onset age. URTI, upper respiratory tract infection.

*IFIH1* encodes a RIG‐I‐like cytoplasmic sensor of long dsRNA and plays a major role in innate immune recognition of RNA viruses. The association analysis between the occurrence of *IFIH1* mutations and GPP onset was statistically significant (*p* < 0.05), indicating a correlation (Table 1). Retrospective analysis of the clinical data from these 80 patients revealed a correlation between the prevalence of URTI and the presence of the *IFIH1* variant locus. There was a total of 70 patients in the *IFIH1* non‐variant group, among which, 21 patients had URTI as the trigger, with an infection rate of 30%. However, in the *IFIH1* variant group of 10 patients, 7 patients had URTI, with an infection rate of 70%; this is significantly higher than the non‐variant group (*p*<0.01) (Figure 2a and Tables 2 and S5). In the *IFIH1* variant group, there were five cases with a disease age of onset of less than 1 year (50%), among which, three cases had URTI and one case had hypoalbuminemia. Additionally, three cases had disease onset between the ages of three and 6 years, and three cases had disease onset over the age of 6 years. In the *IFIH1* non‐variant group, there were 23 cases with an onset age of less than 1 year, accounting for 32.86% (23/70) of the cases. Interestingly, in both the *IFIH1* variant and non‐variant groups, the number of cases with an onset age of less than 1 year was higher compared to the other age groups, but there was no statistically significant difference between those with or without *IFIH1* variants in terms of the number of cases with an onset age less than 1 year (Figure 2b and Table S6). These results suggest that infancy is a high‐risk period for GPP. This may be related to the incomplete establishment of the infant's immune system, making them more susceptible to infections.

**Table 2:** The characteristics of 10 GPP with *IFIH1* variant.

Patient ID	Sex	Ethnicity	Onset age	Clinical presentation	*IFIH1* Nucleotide variations
GPP1	F	Chinese	1 y	URTI	het c.229 C > T
GPP2	M	Chinese	2m	URTI	het c.1041 A > C
GPP3	M	Chinese	7 y 1 m	/	het c.1093 A > G
GPP4	M	Chinese	1 y	URTI	het c.1093 A > G
GPP5	F	Chinese	5 y	URTI	het c.1093 A > G
GPP6	M	Chinese	10 y	URTI	het c.1590 C > G
GPP7	M	Chinese	5 y 11 m	/	het c.1899G > T
GPP8	M	Chinese	5 y 4 m	URTI	het c.2115 A > C
GPP9	F	Chinese	15 d	hypoalbuminemic	het c.2232 T > A
GPP10	F	Chinese	20 d	URTI	het c.2601 G > C

Abbreviations: d, day; het, heterozygotes; m, month; URTI, upper respiratory tract infection; y, year.

### 3.3 Variants in *IFIH1*

*IFIH1* encodes MDA5, which contains a DExD/H‐box RNA helicase domain and a C terminal domain (CTD), both responsible for RNA binding. In addition, MDA5 has two N‐terminal CARDs. The key functional site of MDA5 is the helicase and CARD domain, which are responsible for binding to dsRNA and interacting with the MAVS. According to the bioinformatics prediction analysis, the *IFIH1* variant sites p.L347F and p.K365E located in the helicase ATP‐binding functional domain had the highest predicted pathogenicity at 70% and 71%, respectively. The variant sites p.R705S and p.F744L located in the helicase C‐terminal functional domain had predicted pathogenicity of 61% and 57%, respectively. The CARD1 functional domain was predicted to have a pathogenicity of 22% due to its incomplete protein structure. The non‐functional region variant site p.Glu633Asp was predicted to have a pathogenicity of 35%, while the non‐functional region variant site p.N530K and the RLR CTR region variant site p.E867D were predicted to have lower pathogenicity, at only 4%. (Figure 1b and Table 3). We speculate that these missense variations located at different domains are related to the development of the GPP.

**Table 3:** The pathogenicity prediction of rare *IFIH1* variations.

Position	*IFIH1* variations	*IFIH1* amino acid variations	exon	Domains	Sequence conservation‐a	Protein stability change‐b	Deleterious versus all algorithms‐c
chr2:163174589	c.229 C > T	p.R77W	1	CARD1	*	*	5:23 (0.22)
chr2:163144699	c.1041 A > C	p.L347F	5	Helicase ATP‐binding	9	Destabilizing (−2.034)	16:23 (0.7)
chr2:163144647	c.1093 A > G	p.K365E	5	Helicase ATP‐binding	8	Stabilizing (0.127)	17:24 (0.71)
chr2:163136557	c.1590 C > G	p.N530K	8	none	1	Stabilizing (0.506)	1:23 (0.04)
chr2:163134070	c.1899 G > T	p.E633D	10	none	7	Destabilizing (−1.741)	8:23 (0.35)
chr2:163133386	c.2115 A > C	p.R705S	11	Helicase C‐terminal	6	Destabilizing (−0.316)	14:23 (0.61)
chr2:163133269	c.2232 T > A	p.F744L	11	Helicase C‐terminal	8	Destabilizing (−0.668)	13:23 (0.57)
chr2:163128751	c.2601 G > C	p.E867D	13	RLR CTR	1	Destabilizing (−0.301)	1:23 (0.04)

*Note:* ^a^The conservation scores of this site (9‐conserved, 1‐variable), calculated by ConSurf. ^b^Protein stability change upon mutation was computed by DUET, scores are listed in brackets, the unit is kcal/mol. ^c^The pathogenicity of mutations were predicted by VarCards, an integrated genetic and clinical database for coding variants in the human genome, the scores were listed in brackets.

* There is not show the data of 229 site in the Sequence conservation database.

**Figure 3:**
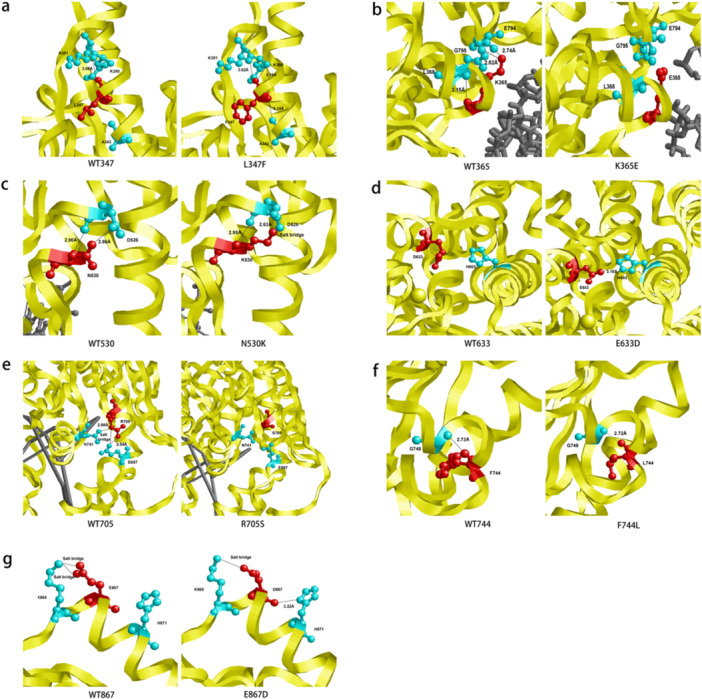
The protein structure of MDA5. The MDA5 protein structure was analyzed using the resolved crystal structure data as a template (PDB code: 4GL2). The MDA5 structure after amino acid mutation was constructed by Swiss‐Model. Molecular forces among the amino acid residues of the proteins were analyzed by LigPlot + and visualized by RasMol. The MDA5 structure is shown in yellow cartoon images, with red sticks representing MDA5 WT and variants, cyan sticks representing amino acids bound to wild‐type and mutant, and gray bars representing double‐stranded RNA. (a) After L347F mutation, it forms a hydrogen bond with lysine 351 (length changes, and can form a hydrogen bond with alanine 343 (bond length 3.14 A) and a hydrogen bond with lysine 350 (bond length 3.16 A); (b) Lysine at 365 position of WT can form hydrogen bonds with amino acids at position 368,794 and 795 respectively (bond length is 3.15 A, 2.74 A, 2.82 A respectively); when K365E is mutated to glutamate, it cannot form hydrogen bonds with the surrounding protein, and the double‐stranded RNA structure it binds is changed. (c) After the N530K mutation, the hydrogen bond position and bond length formed by the binding of the aspartate at position 526 are changed. (d) After E633D mutation, the hydrogen bonds formed between histidine and the position 603,629 phenylalanine and lysine at positions 636 and 637 are altered. (e) After the R705S mutation, it fails to bind to glutamate at position 697 and aspartate at position 741, thus affecting the binding of the protein to double‐stranded RNA. (f) After the F744L mutation, the hydrogen bond length formed with the glycine at position 748 becomes shorter after the F744L mutation. (g) After the E867D mutation, the salt bridge formed by the lysine at position 865 changes from one to two, forming a new hydrogen bond with the histidine at position 871. *Note:* The protein structure of MDA5 (PDB code: 4GL2) does not contain the R77 site, so it is impossible to analyze whether the R77W mutation site affects the protein structure.

To investigate whether the variants are deleterious, bioinformatic programs were first used to analyze the pathogenicity of rare variants in the *IFIH1* (Table 3). The conservation predictions by ConSurf indicated that the rare variants p.L347F, p.K365E, p.N530K, p.E633D, p.R705S, p.F744L in *IFIH1* were conserved. Protein stability change upon mutation was computed by DUET. The results indicated that the variants p.K365E and p.N530K were stabilizing. The variant p.R77W could not be predicted by ConSurf and DUET because this site is not included in the protein structure. The pathogenicity of the variants was predicted by VarCards. The results indicated that all eight variants, except p.N530K and p.E867D, were deleterious, potentially causing changes in protein structure and function. Analysis of the protein structures by Swiss‐Model revealed that some *IFIH1* variants had changed molecular forces between the amino acid residues, causing changes in their protein structures that may lead to protein loss‐of‐function (Figure 3). The amino acids of the sites at 347, 365, 705, and 744 were mutated, the structures of the amino acids were changed, and the hydrogen bond positions and bond lengths formed by the binding of these amino acids were also modified. In particular, the mutation of sites 365 and 705 had effects on binding between the double‐stranded RNA structure and the protein. While mutations at sites 530, 633, and 867 had slight effects on the structures of the amino acids. When comparing the conservation of amino acids between different species, the results showed that the positions 347, 365, 705, 744, and 867 were highly conserved (Figure S1).

### 3.4 Functional Characterization of *IFIH1* Variants

**Figure 4:**
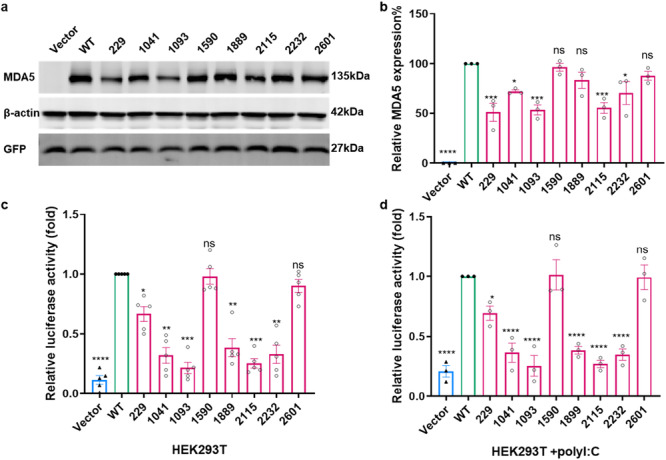
Functional characterization of the *IFIH1* variants. (a) Expression levels of the protein MDA5 coding by *IFIH1* isoforms are shown under the plot in the Western blot gel. HEK293T cells were transfected with *IFIH1* WT or variants expression plasmids (3 µg) and pEGFP (200 ng) in six well cell plates. Cells were collected at 24h post transfection. (b) The statistic of the MDA5 expression in wild type and variants. The expression levels were analyzed for relative *IFIH1* WT expression. Data are shown as means ± standard deviation (SD) and analyzed by one‐way ANOVA followed by Dunnett's test. (*n* = 3, *p < 0.05, **p < 0.01, ***p < 0.001, ****p < 0.0001.) (c) and (d) IFN‐β reporter assays of MDA5 and its point mutants. HEK293T cells were transfected with ‐luciferase reporter plasmids p4‐IFN‐β promoter (100 ng) and p3Rluc (30 ng) and *IFIH1* WT or variants expression plasmids (25 ng) in 48 well plate. At 6 h post transfection, cells were either mock transfected or transfected with a synthetic double‐stranded RNA control poly(I:C) (10 µg/mL). At 18h after poly(I:C) transfection (24 h after plasmid DNA transfection), cells were collected and analyzed for dual luciferase activity. The results are expressed as relative IFN‐β promoter activity. Error bars, standard deviations calculated from more than three independent experiments (mean ± SD, *n* ≥ 3). The statistic was analyzed using one‐way ANOVA followed by Dunnett's test (*p < 0.05, **p < 0.01, ***p < 0.001, ****p < 0.0001.).

Next, to determine whether variants of the *IFIH1* affect its protein expression, co‐transfection of expression plasmids containing the wild‐type or mutant coding sequences of *IFIH1*, along with a pEGFP expression plasmid as a transfection control, was performed. Immunoblotting was used to detect the expression of *IFIH1* in the cells. The results revealed that compared to the wild‐type, transfection of mutant plasmids 229, 1041, 1093, 2115, and 2232 resulted in a significant decrease in MDA5 protein expression (Figure 4a,b). After mutation at site 1899, the protein expression level exhibited a slight decrease compared to the wild type, but this difference was not statistically significant.

To further explore whether *IFIH1* mutation is a loss‐of‐function variant, a dual luciferase reporter assay was used to test the effects of *IFIH1* variants on IFN‐β promoter activity. The effects of the wild‐type and mutant *IFIH1* on the type I interferon pathway were tested in HEK293T cells. The dual luciferase results showed that variants 229, 1041, 1093, 1899, 2115, and 2232 had significant inhibitory effects on IFN‐β promoter activity, while 1590 and 2601 had little effects on IFN‐β promoter activity (Figure 4c). After stimulation with poly (I:C) (synthetic analog of dsRNA), the various mutation sites exhibited similar inhibitory effects on IFN‐β promoter activity (Figure 4d). This indicates that these six variants are loss‐of‐function mutations and may impair the activation of MAVS and interferon production, resulting in impaired innate immune defense. In conclusion, the mutations of the *IFIH1* resulted in reduced production of IFN‐β compared to the wild‐type, both with and without the poly(I:C).

Collectively, these findings demonstrate that six out of the eight variants identified in our study population lead to severe disruption of *IFIH1* signaling function, enzymatic activity, and protein stability in vitro. Additionally, the finding that the mutant *IFIH1* isoforms interfere with the wild‐type protein in terms of IFN‐β induction and protein stability suggests a dominant negative role of heterozygous loss‐of‐function variants in *IFIH1*.

## 4 Discussion

This study employed whole exome sequencing and identified eight variants in *IFIH1* in 10 patients with GPP. The eight variants discovered were overexpressed and tested for their ability to drive luciferase expression from an IFN‐β promoter after stimulation with intracellular poly(I:C). Six variants were found to decrease the activity of the IFN‐β promoter. GPP patients carrying *IFIH1* mutations exhibited classic clinical features, diffuse erythema, pustules, and plaques (Figure S2). There were no significant differences observed compared to non‐carriers.

GPP is a heterogeneous disease with a wide spectrum of disease severity and a highly variable clinical course. GPP can occur independently or develop from existing psoriasis; its course is typically characterized by recurrent episodes and a poor prognosis. The etiology and pathogenesis of GPP are not fully understood but may be related to factors such as genetics, infection, immune abnormalities, and endocrine disorders.

While this study provides novel insights into the genetic basis of GPP by identifying *IFIH1* variants and their potential interactions with other GPP associated genes, several limitations warrant acknowledgment: First, the cohort size in this study was relatively small, particularly with only 10 GPP patients analyzed for *IFIH1* variants. This limited sample size may reduce statistical power, potentially obscures some genetic associations, and necessitates validation in larger cohorts to confirm the significance of the findings. Second, the functional validation primarily relied on an in vitro reporter gene assay, which confirmed the impact of specific *IFIH1* variants on IFN‐β promoter activity. However, the precise molecular mechanisms by which these variants contribute to GPP pathogenesis remain unclear. Further investigation is required to elucidate their effects on downstream signaling pathways and cellular responses. Psoriasis is significantly associated with other systemic inflammatory diseases, and the core mechanism of their shared pathogenic pathways lies in chronic inflammation and the pivotal role of pro‐inflammatory cytokines [30]. Third, due to the retrospective nature of the study, pathogen data for some patients were not systematically documented. This incomplete infection history may compromise the accuracy of the etiological association analysis. Finally, in vitro functional assays cannot fully recapitulate the complex immune microenvironment of GPP. Future studies should employ in vivo models to validate our findings.

This study establishes an association between *IFIH1* loss‐of‐function variants and infection‐triggered generalized pustular psoriasis (GPP), expanding the previous understanding of pathogenesis dominated by *IL36RN* and *CARD14*. Compared to the *IL36RN* mutation rates (28.8%–66.7%) reported in previous studies [31], the *IFIH1* variant frequency (12.5%) observed in our cohort suggests it may be a relatively common susceptibility gene. Mechanistically, we observed that the type I interferon deficiency resulting from *IFIH1* variants exhibits a synergistic effect with impaired *AP1S3*‐TLR3 signaling, both pointing to defective antiviral defense as a central element in infection‐triggered GPP [32]. Notably, the *IFIH1* variants identified here differ from the gain‐of‐function mutations associated with Aicardi‐Goutières syndrome and Singleton‐Merten syndromes [33, 34]. This inverse genotype‐phenotype correlation offers a novel perspective for understanding the pleiotropy of MDA5.

**Figure 5:**
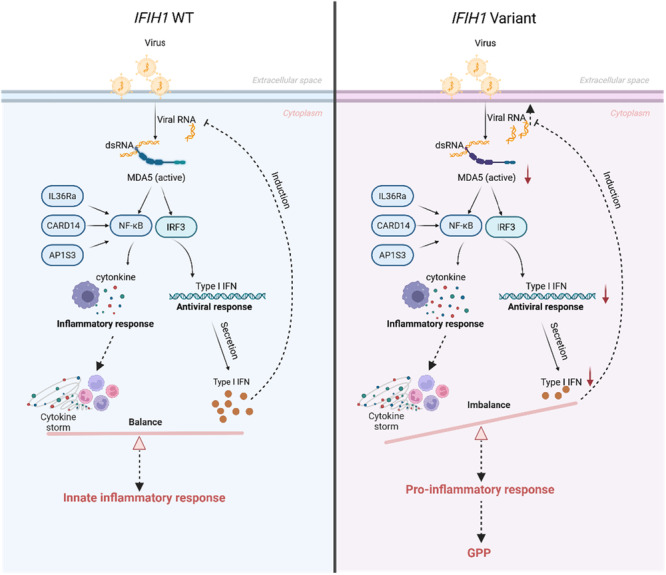
The mechanisms of MDA5 in GPP. Thick, red, solid lines: the key mechanistic steps validated by our experimental results; thin, black, solid lines: well‐established in the literature and provide context for our model; black dashed lines: speculative or hypothetical connections that form the basis for future research questions. The possible mechanisms include structural alterations or reduced expression of the MDA5 protein encoded by the *IFIH1*, leading to diminished function and reduced activation of the transcription factors IRF3, IRF7, and NF‐κB. This results in reduced expression of type I interferons, which weakens antiviral capabilities, allowing common upper respiratory viruses such as rhinovirus or RSV to proliferate and induce systemic inflammation. This exacerbates local tissue damage, and these viruses or pro‐inflammatory cytokines may enter the systemic circulation and reach the skin to trigger inflammation in skin. Moreover, due to defects in anti‐inflammatory genes such as *IL36RN*, which is primarily expressed in the skin, or enhanced pro‐inflammatory effects resulting from variants in pro‐inflammatory genes such as *CARD14*, the balance between pro‐inflammatory and anti‐inflammatory responses in the skin shifts towards pro‐inflammation, leading to the onset of GPP.

Based on the current findings and the available literature, we hypothesize that loss‐of‐function *IFIH1* variants collaborate with variants in genes such as *IL36RN*, contributing significantly to the pathogenesis of GPP triggered by URTI. Potential mechanisms involve structural changes or reduced expression of the MDA5, impairing its function and reducing activation of IRF3, IRF7, and NF‐κB. This leads to decreased type I interferon production, compromising antiviral responses. Consequently, viruses can proliferate, triggering systemic inflammation. This worsens local damage, and viruses or pro‐inflammatory cytokines may enter the systemic circulation, reaching the skin and initiating inflammation. Furthermore, defects in *IL36RN* or *CARD14* or *AP1S3* shift the skin's inflammatory balance towards pro‐inflammation, leading to GPP onset. (Tables S7 and S8) (Figure 5). COVID‐19 virus is a potential trigger for a variety of autoimmune and autoinflammatory skin diseases. Generalized pustular psoriasis (GPP), as a severe autoinflammatory disorder, has been reported in several cases to experience acute flares or exacerbations following COVID‐19 infection.COVID‐19 infection can induce a massive cytokine storm, characterized by significantly elevated levels of IL‐1β, IL‐6, and TNF‐α. These pro‐inflammatory cytokines can stimulate keratinocytes to secrete IL‐36 [35, 36]. In individuals with genetic susceptibility (such as mutations in IL36RN), this surge in IL‐36 cannot be properly inhibited, leading to extensive neutrophil accumulation in the skin and the formation of pustules.

In this cohort of 80 pediatric GPP patients, whole‐exome sequencing revealed that 21.25% (*n* = 17) of cases lacked detectable pathogenic variants in known GPP‐associated genes (*IL36RN*, *CARD14*, *AP1S3, MPO*, *SERPINA3*), the genetic heterogeneity in GPP, where a subset of patients remains genetically uncharacterized despite advancements in gene discovery. Thus, it is speculated that there may be other gene variations associated with the onset of GPP that are yet to be discovered.

In pediatric GPP patients, infection (especially URTI) is the primary and most common trigger, with a significantly higher proportion than in adults. Moreover, childhood‐onset GPP is often more closely associated with genetic mutations and typically occurs without a prior history of psoriasis vulgaris. In contrast, adult patients, due to a higher prevalence of comorbidities, are more frequently affected by drug‐induced GPP compared to children. In addition, factors such as pregnancy and hormonal changes, emotional stress, excessive fatigue, and lifestyle habits like smoking and alcohol consumption contribute to a certain proportion of adult‐onset GPP but are relatively rare in children. This is closely related to the fact that the immune system in children is still developing, making them more sensitive to pathogen detection and response [37, 38, 39]. Palmoplantar pustulosis (PPP) is one of major subtypes of localized pustular psoriasis (PP). Although both GPP and PPP are characterized by sterile pustules, they exhibit significant differences in clinical manifestations, disease course, and genetic background. Genetic testing aids in distinguishing GPP from PPP, providing molecular‐level diagnostic evidence, particularly for clinically atypical cases. Genetic heterogeneity determines the essential differences in the pathogenesis of GPP and PPP. Genetic testing‐assisted diagnosis can more accurately identify disease subtypes and avoid misdiagnosis. Notably, biologics targeting the IL‐36 pathway are highly effective for GPP but have limited efficacy for PPP [1, 40].

The progressive elucidation of the genetic mechanisms underlying GPP has significantly advanced personalized targeted therapies. Previously, GPP treatment primarily focused on symptomatic relief, lacking precise targets, resulting in limited efficacy and numerous adverse reactions. Compared with conventional pharmacotherapy for pediatric GPP, biologics demonstrate superior clinical efficacy, as reflected by markedly reduced hospitalization duration and accelerated pustule resolution, coupled with a more favorable safety profile [41]. This study's analysis of GPP genetic mechanisms and inducing factors offers novel insights and feasible intervention strategies for the clinical prevention and treatment of pediatric GPP. Recurrent episodes are a prominent feature of pediatric GPP. Frequent exacerbations not only cause physical suffering but may also impact growth, development, and psychological well‐being, necessitating targeted preventive and interventional measures [38]. For children with a clinical diagnosis of GPP, genetic screening is recommended to identify underlying defects. In those found to have such defects and a history of recurrent GPP, early antiviral treatment at the onset of an upper respiratory tract infection may help inhibit viral triggers, reduce the risk of GPP flares, and minimize recurrence.

## 5 Conclusion

In this study, we identified rare *IFIH1* variants in children with GPP through whole exome sequencing. Retrospective analysis of clinical data indicated that children with GPP had a history of URTI preceding the onset of GPP. These findings suggest a potential interplay between *IFIH1* variants and variations in *IL36RN* or other gene polymorphisms, which may contribute to broader insights into the pathogenesis of GPP triggered by URTI.

## Author Contributions

Yaqin Liu contributed to data curation, formal analysis, methodology, and writing the original draft. Yanan Sun performed methodology, data curation, and formal analysis. Juan Yang contributed to data curation, formalanalysis. Hongmei Li secured funding acquisition, contributed to writing the original draft, and provided resources. Weihui Zhou secured funding acquisition, contributed to writing the original draft, review, and editing, provided resources and supervision, and conceptualized the study. Shasha Meng conceptualized the study, secured funding acquisition, contributed to writing the original draft, review, and editing, provided resources and supervision, and performed data curation and investigation.

## Ethics Statement

This study involving human participants was reviewed and approved by the Ethics Committee of Children's Hospital of Chongqing Medical University. The approval reference numbers are 2018‐35 and 2023‐23.

## Consent

Written informed consent was obtained from all participants prior to their inclusion in this study. For participants under 18 years of age or those unable to consent, written assent was obtained alongside informed consent from their legally authorized representative.

## Conflicts of Interest

The authors declare no conflicts of interest.

## Supporting information


**Figure S1:** The conservation of each amino acid across various vertebrate species. **Figure S2:** Clinical presentationof the patient on admission.

**Table S1:** The sequence of primers. **Table S2:** Clinical features of GPP enrolled in the study. **Table S3. Table S4:** The proportion of the GPP with IFIH1 variants. **Table S5:** The proportion of URTI. **Table S6:** The proportion of one‐set age. **Table S7:** The variation of related gene in GPP. **Table S8:** The Function of GPP Relative Gene.

## Data Availability

The raw whole‐exome sequencing data are now available from the corresponding author upon reasonable request in the Genome Sequence Archive (GSA) under accession number HRA015036 (GSA: https://ngdc.cncb.ac.cn/).
