# Supplementary material for: Repeated Episodes of GPP Induced by Respiratory Infections Are Mediated by Loss‐of‐Function IFIH1
Source: Immun Inflamm Dis. 2026 Mar 18;14(3):e70397. doi: 10.1002/iid3.70397 (PMC13097489; doi:10.1002/iid3.70397)
Supplement: Supplementary file 1 — Figure S1: The conservation of each amino acid across various vertebrate species. Figure S2: Clinical presentationof the patient on admission. [file IID3-14-e70397-s002.docx]

| Table S1. The sequence of primers | | |
| --- | --- | --- |
| Primer | Seqence(5'to3') | bases(bp) |
| g229F | GCAAACGCACAAGGAAGC | 18 |
| g229R | CAGGCACAGAGCGGTAGA | 18 |
| g1041-1093F | AAACAATATTTGATCCTTGATTCCA | 25 |
| g1041-1093R | TGTTGCTCCTTCCAGTCC | 18 |
| g1590F | CGAACTGTCATAACCCCTCC | 20 |
| g1590R | CAAATGTTGCTGATTGTGGG | 20 |
| g1899F | CTGAATGACCAAATGACTGA | 20 |
| g1899R | GGAAATCGCAAAGAACG | 17 |
| g2115-2232F | GACAAGCCAGGACAACATAGT | 21 |
| g2115-2232R | GTCAGTCATTTGGTCATTCAGT | 22 |
| g2601F | GATAATTTCCTGCCCACCA | 19 |
| g2601R | AAGACGACTCCTCAGTCCAAG | 21 |
| p229F | ACTtGGGAATTCGTGGAGGCC | 21 |
| p229R | CCAACCAAGGTGCCAGACTC | 20 |
| pl041F | CTTcGACAAGAAGAAAAAAGCATCTGAGC | 29 |
| p1041R | TGATCCTTGGCAATGTAAACAGC | 23 |
| p1093F | TACTGCTAGTTGAACAG | 17 |
| p1093R | CCTcATTGACAAGAACTAT | 19 |
| p1590F | AAAAAcCAAATACAGGAGCCATG | 23 |
| p1590R | CAGTTGATCAAGGTTTTCTTTAAC | 24 |
| pl899F | AAGAtAAAGATAAGAAGTTTGCAG | 24 |
| p1899R | CATTATAGAAAGTTTCAAGATGAG | 24 |
| p2115F | AAGcAATACCATAATGGAGC | 20 |
| p2115R | AATTTGGTCAGCTTTTCATT | 20 |
| p2232F | ATTaGCTGAAGTAGGAGTCAAAGC | 24 |
| p2232R | TTTTCATTTTCAGTAATCCACTGG | 24 |
| p2601F | AGAcGAGTATGCTCATAAGA | 20 |
| p2601R | GGTTTCATATTTTGAACACAATG | 23 |

| Table S2. Clinical features of GPP enrolled in the study | | |
| --- | --- | --- |
| Characteristic | GPP(n=80) | % |
| Male | 48 | 60 |
| female | 32 | 40 |
| mean age of GPP onset, years | 3.9 | / |
| erythema | 80 | 100 |
| aseptic pustules | 80 | 100 |
| arthralgia and asthenia | 40 | 50 |

| Table S4. The proportion of the GPP with *IFIH1* variants | | | | | | |
| --- | --- | --- | --- | --- | --- | --- |
| Variant ID | Position | *IFIH1*variations | Number of GPP  with *IFIH1* variations | Number of CON*  with *IFIH1* variations | Number of GPP | Average number of CON |
| rs147278787 | chr2:163174589 | c.229C>T | 1 | 69 | 80 | 9864 |
| rs183412282 | chr2:163144699 | c.1041A>C | 1 | 21 | 80 | 9864 |
| rs117608083 | chr2:163144647 | c.1093A>G | 3 | 143 | 80 | 9864 |
| rs369245661 | chr2:163136557 | c.1590C>G | 1 | 1 | 80 | 9864 |
| rs550930413 | chr2:163134070 | c.1899G>T | 1 | 63 | 80 | 9864 |
| rs185928139 | chr2:163133386 | c.2115A>C | 1 | 80 | 80 | 9864 |
| rs201142986 | chr2:163133269 | c.2232T>A | 1 | 67 | 80 | 9864 |
| rs764489663 | chr2:163128751 | c.2601G>C | 1 | 9 | 80 | 9864 |
| Total |  |  | 10 | 453 | 12.5%(10/80) | 4.59%(453/9864) |

GPP: generalized pustular psoriasis(n=80); CON: control;

Differences in frequencies of *IFIH1* variants between groups were analyzed by Fisher exact tests.

*The control data is the population numbers corresponding variants in the east Asian population adopted from Genome Aggregation Database (https://gnomad.broadinstitute.org/).

| Table S5. The proportion of URTI | | | | | |
| --- | --- | --- | --- | --- | --- |
|  | URTI | without URTI | *OR* | 95%*CI* | *P* value |
| GPP with *IFIH1* variant | 7(70%) | 3(30%) | 5.44 | 1.26-20.36 | 0.028 |
| GPP without *IFIH1* variant | 21(30%) | 49(70%) | 0.18 | 0.05-0.80 |  |

URTI: upper respiratory tract infection；*OR*: odds ratio; *CI*: confidence interval.

Differences in GPP with or without *IFIH1* variants between groups were analyzed by Fisher exact tests.

| Table S6. The proportion of one-set age | | | | | | |
| --- | --- | --- | --- | --- | --- | --- |
|  | 0-1year | 1-3year | 3-6year | 6-18year | Chi-squaare | *P* value |
| GPP with *IFIH1* variant | 5(50%) | 0(0) | 3(30%) | 2(20%) | 3.72 | 0.29 |
| GPP without *IFIH1* variant | 23(32.86%) | 16(22.86%) | 16(22.86%) | 15(21.43%) |  |  |

| Table S7. The variation of related gene in GPP | | | | | | |
| --- | --- | --- | --- | --- | --- | --- |
| Patient ID | *IFIH1* Nucleotide variations | *IL36RN* Nucleotide variations | *CARD14* Nucleotide variations | *SERPINA3* Nucleotide variations | *AP1S3* Nucleotide variations | *MPO* Nucleotide variations |
| GPP1 | het c.229C>T | het c.115+6T>C | N | N | N | N |
| GPP2 | het c.1041A>C | N | N | N | N | N |
| GPP3 | het c.1093A>G | N | N | het c.1240A>G | N | N |
| GPP4 | het c.1093A>G | hom c.115+6T>C | N | N | N | N |
| GPP5 | het c.1093A>G | het c.115+6T>C | het c.2192C>T | N | N | N |
| GPP6 | het c.1590C>G | hom c.115+6T>C | N | het c.923T>C | N | N |
| GPP7 | het c.1899G>T | hom c.115+6T>C | N | N | N | N |
| GPP8 | het c.2115A>C | N | N | N | N | N |
| GPP9 | het c.2232T>A | het c.115+6T>C | N | N | N | N |
| GPP10 | het c.2601G>C | hom c.115+6T>C | het c.746A>C | het c.1240A>G | N | N |

het:heterozygotes; hom:homozygotes; N: no variation

| Table S8. The Function of GPP Relative Gene | | | |
| --- | --- | --- | --- |
| **Gene Symbol** | **Protein** | **Function** | **Reference** |
| *IL36RN* | Interleukin 36 Receptor Antagonist (IL-36Ra) | Anti-inflammatory regulator; inhibits IL-36 signaling. Mutations cause GPP. *IL36RN* mutations lead to overactivation of the IL-36 signaling pathway, inducing excessive expression of neutrophil chemo-attractants such as IL-6 and IL-8. This promotes the migration and aggregation of neutrophils in the epidermis, leading to the formation of abscesses and inflammatory damage, thereby promoting GPP. | 9-11 |
| *CARD14* | Caspase Recruitment Domain Family Member 14 | Scaffold protein activating NF-κB pathway. Gain-of-function mutations linked to psoriasis and GPP.  Mutations in *CARD14* promote the activation of the NF-κB signal within keratinocytes which, in turn, promotes the transcription of pro-inflammatory cytokines and contributes to the onset of the disease in affected individuals. | 12-14 |
| *AP1S3* | Adaptor Related Protein Complex 1 Subunit Sigma 3 | Vesicular trafficking in keratinocytes. Mutations impair autophagy and promote GPP inflammation.  Loss of function in *AP1S3* disrupts the intracellular transport of TLR-3, leading to reduced downstream expression of IFN-β, increased expression of pro-inflammatory cytokines such as IL-1, and excessive expression of IL-36α, thereby contributing to the development of skin inflammation. | 15,16 |
| *MPO* | Myeloperoxidase | Neutrophil enzyme producing hypochlorous acid . Biomarker for neutrophilic inflammation. In neutrophils lacking *MPO*, the enzyme activity responsible for the activation of IL-36α, IL-36β, and IL-36γ precursors is increased, leading to the characteristic pro-inflammatory imbalance in the IL-36 pathway in GPP. | 17 |
| *SERPINA3* | Serpin Family A Member 3 (Alpha-1-Antichymotrypsin, ACT) | Protease inhibitor regulating inflammation. Overexpressed in GPP skin lesions.  In normal skin tissue, *SERPINA3* inhibits cathepsin-G to regulate the activity of inflammatory factors. Mutations result in the overactivation of the pro-inflammatory factor IL-36β, amplifying the inflammatory response. | 18,19 |


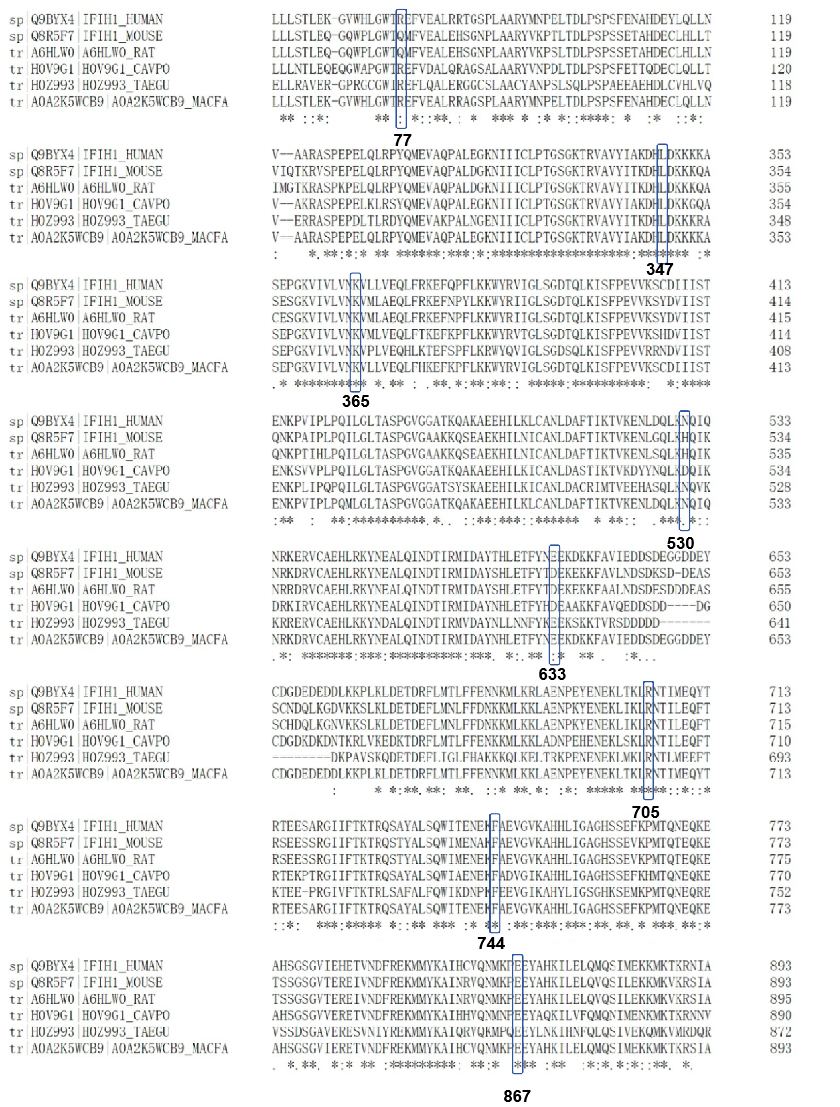


Figure S1. The conservation of each amino acid across various vertebrate species

The align results showed the high conservation at the site of p.L347, p.K365, p.R705, p.F744, p.E867.


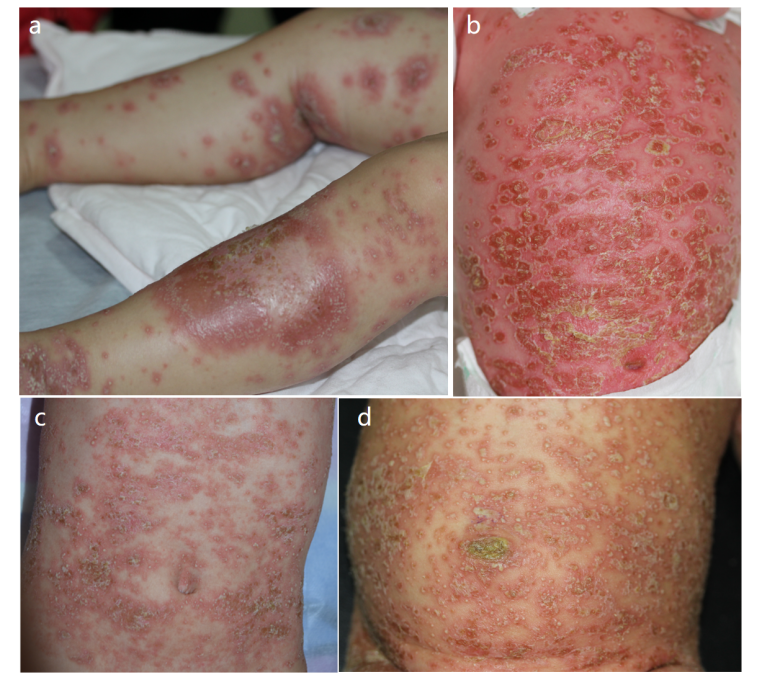


Figure S2. Clinical presentation of the patient on admission.

a.GPP patient with *IFIH1* c.2232 T>A. multiple pustules, partly coalesce into lakes of pus over the widespread edematous erythematous patches on the lower limbs. Female, Age at this visit: 2years 6months old.

b.GPP patient with *IFIH1* c.2601 G>C, extensive erythematous patches studded with multiple pustules, crusts on the abdomen. Female, Age at this visit: 20days old.

c.GPP patient without *IFIH1* variant, extensive erythematous patches studded with multiple pustules, crusts and scales on the abdomen. Female, Age at this visit: 2 years 8months old.

d.GPP patient without *IFIH1* variant, extensive erythematous patches studded with multiple pustules, partly coalesce into lakes of pus on the abdomen.Female, Age at this visit: 1months old.
